# Morphometric Analysis of the Eye by Magnetic Resonance Imaging in *MGST2-*Gene-Deficient Mice

**DOI:** 10.3390/biomedicines12020370

**Published:** 2024-02-05

**Authors:** Chaomulige, Toshihiko Matsuo, Kohei Sugimoto, Mary Miyaji, Osamu Hosoya, Masashi Ueda, Ryosuke Kobayashi, Takuro Horii, Izuho Hatada

**Affiliations:** 1 Graduate School of Interdisciplinary Science and Engineering in Health Systems, Okayama University, Okayama 700-8558, Japan; sugimotokohei@s.okayama-u.ac.jp; 2 Department of Ophthalmology, Okayama University Hospital, Okayama 700-8558, Japan; 3 Department of Medical Neurobiology, Graduate School of Medicine, Dentistry, and Pharmaceutical Sciences, Okayama University, Okayama 700-8558, Japan; mmiyaji@okayama-u.ac.jp (M.M.); hosoya@okayama-u.ac.jp (O.H.); 4 Department of Biofunctional Imaging Analysis, Graduate School of Medicine, Dentistry, and Pharmaceutical Sciences, Okayama University, Okayama 700-8530, Japan; mueda@cc.okayama-u.ac.jp; 5 Biosignal Genome Resource Center, Institute for Molecular and Cellular Regulation, Gunma University, Maebashi 371-8512, Japan; rkobayashi@gunma-u.ac.jp (R.K.); horii@gunma-u.ac.jp (T.H.); hatada@gunma-u.ac.jp (I.H.); 6 Viral Vector Core, Gunma University Initiative for Advanced Research (GIAR), Maebashi 371-8511, Japan

**Keywords:** comitant strabismus, *MGST2* gene, mouse models, genetics, CRISPR/Cas9, PCR, MRI, eye morphology, neuro-ophthalmology

## Abstract

Strabismus, a neuro-ophthalmological condition characterized by misalignment of the eyes, is a common ophthalmic disorder affecting both children and adults. In our previous study, we identified the microsomal glutathione *S*-transferase 2 (*MGST2*) gene as one of the potential candidates for comitant strabismus susceptibility in a Japanese population. The *MGST2* gene belongs to the membrane-associated protein involved in the generation of pro-inflammatory mediators, and it is also found in the protection against oxidative stress by decreasing the reactivity of oxidized lipids. To look for the roles of the *MGST2* gene in the development, eye alignment, and overall morphology of the eye as the possible background of strabismus, *MGST2* gene knockout (KO) mice were generated by CRISPR/Cas9-mediated gene editing with guide RNAs targeting the *MGST2* exon 2. The ocular morphology of the KO mice was analyzed through high-resolution images obtained by a magnetic resonance imaging (MRI) machine for small animals. The morphometric analyses showed that the height, width, and volume of the eyeballs in *MGST2* KO homozygous mice were significantly greater than those of wild-type mice, indicating that the eyes of *MGST2* KO homozygous mice were significantly enlarged. There were no significant differences in the axis length and axis angle. These morphological changes may potentially contribute to the development of a subgroup of strabismus.

## 1. Introduction

Strabismus refers to the inability of both eyes to focus on the target at the same time, which is frequently associated with reduction or loss of binocularity [1,2,3,4,5]. The causes of strabismus are varied but are usually due to a lack of coordination among extraocular muscle movements [2]. In addition, several studies indicate that different regions of the brain, including the brainstem, pons, cerebellum, cortex, visual pathways, and neurons, are associated with abnormal eye alignment and eye movement [6,7]. These findings suggest complicated processes of eye coordination. Strabismus can be divided into two main types as comitant strabismus and incomitant strabismus [1,2,3,4,5]. In comitant strabismus, the misalignment remains relatively constant regardless of the direction of gaze, and the common forms include esotropia and exotropia. Incomitant strabismus presents misalignment that varies with the direction of gaze and is caused by muscle imbalance or nerve issues [2]. Comitant strabismus is generally considered to be more common than incomitant strabismus [1,2,3,4,5].

Comitant strabismus is often seen in childhood, and it can manifest early in life or develop later. The clinical features are highly heterogeneous and largely influenced by genetic factors, such as family histories and twin consistency, as well as various prenatal and early life environmental factors, such as prematurity and poor health during pregnancy. Both genetic factors and environmental factors increase the risk of strabismus [8,9]. Since Hippocrates first observed familial strabismus, interest in genetic risk factors has increased [4]. Multiple studies have been conducted using various approaches to investigate genetic causation, which led to a growing understanding of the factors that contribute to comitant strabismus [10]. However, the exact cause can be complex and unclear, highlighting the need for different approaches to clarify and understand this prevalent disorder. In our previous research, we identified the susceptibility loci in 4q28.3 regions for comitant strabismus that comprised both esotropia and exotropia in Japanese families, and then we conducted single-nucleotide polymorphism (SNP) analyses to narrow the chromosomal loci down to a single gene in Japanese and U.S. databases. We used three different linkage analyses and found that single-nucleotide polymorphisms (SNPs) with significant *p*-values were present only on the microsomal glutathione S-transferase 2 (*MGST2*) gene on chromosome 4q28.3. Our results suggest that the *MGST2* gene is a potential candidate gene for strabismus susceptibility at the chromosome 4q28.3 locus [11,12].

The *MGST2* gene is a member of the membrane-associated proteins in the eicosanoid and glutathione metabolism (MAPEG) family and plays an important role in cellular detoxification and defense against oxidative stress [13,14]. Its primary function is to catalyze the conjugation of reduced glutathione to electrophilic compounds, thereby facilitating the neutralization and efflux of these compounds. This detoxification process is indispensable for maintaining cellular redox homeostasis and preventing damage caused by reactive oxygen species and toxic molecules [15,16,17]. In addition, several studies have revealed that *MGST2* produces leukotriene C_4_, a key component for intracerebral signaling involved in endoplasmic reticulum (ER) stress, oxidative DNA damage, and cell death [18]. It is indicated that *MGST2* plays a role in regulating ER stress-related pathways.

To determine what role the *MGST2* gene would play in the development, eye alignment, and overall morphology of the eye as possible background of comitant strabismus, we used CRISPR/Cas9 (clustered regularly interspaced short palindromic repeat/CRISPR-associated 9) technology to create *MGST2* knockout (KO) mice, then utilized small animal MRI to capture three-dimensional (3D) images of eye morphology and to employ imaging techniques to visualize the intricate anatomical structures of the eye in 3D. As the design of the study, we first analyzed *MGST2* KO heterozygous mice compared with the wild type (WT) in a pilot study to establish the MRI methods, and we then proceeded to compare the *MGST2* KO homozygous mice with the wild type in a main study.

## 2. Materials and Methods

### 2.1. Animal and Ethics Statement

C57BL/6J mice were purchased from the commercial animal breeder, Charles River Laboratories (Yokohama, Japan). The generation of *MGST2* KO mice in this study was approved by the Animal Care and Use Committee, Gunma University (approval number 17-033). All experiments and breeding of *MGST2* KO mice in this study were approved by the Animal Care and Use Committee, Okayama University (approval number OKU-2021324). Animal management was performed strictly in accordance with the standards of the Department of Animal Resources, Advanced Science Research Center, Okayama University, based on the Act on Welfare and Management of Animals in Japan.

### 2.2. Generation of MGST2 KO Mice

We utilized the CRISPR/Cas9 system with Cas9 endonuclease and guide RNAs (gRNAs) to produce *MGST2* KO mice. The target sequences of gRNAs were ACGACATTCCGGTCCCTTGT**AGG**(*MGST2*L2) and AGGGGAAAGCGTAATACAGA**AGG** (*MGST2*R2), with the protospacer adjacent motif (PAM) sequences underlined. To accomplish this, two gRNAs (1.5 μM each crRNA/tracrRNA in mixture; Integrated DNA Technologies, Coralville, IA, USA) were mixed with recombinant Cas9 protein (100 ng/μL; GeneArt Platinum Cas9 Nuclease, Thermo Fisher Scientific, Waltham, MA, USA) in Opti-MEM I (Life Technologies, Carlsbad, CA, USA). Electroporation was performed as follows [19,20]: The electroporation condition was established by connecting the electrode (LF501PT1–10; BEX, Tokyo, Japan) to the CUY21EDIT electroporator (BEX) under a stereoscopic microscope. Embryos were placed in a line between the electrode gap filled with 5 μL of Cas9/gRNAs mixture. Electroporation was performed by setting at 30 V (3 msec ON + 97 msec OFF) with seven electric pulses.

### 2.3. Genotyping Analysis

The genotypes were confirmed by polymerase chain reaction (PCR) using Q5 Hot Start High-Fidelity DNA Polymerase (M0493S, New England Biolabs, Ipswich, MA, USA) and the following primers: 5′-TTCTAGTAGATAGTCCTGGTACCCAAC-3′ (forward), 5′-CCACCATGCTGAAGAGACAA-3′ (reverse). The PCR amplification conditions were as follows: a first denaturation step (98 °C, 30 s), followed by 30 cycles of denaturation (98 °C, 5 s), annealing (66 °C, 10 s), and extension (72 °C, 10 s), and then a final extension step (72 °C, 2 min). Electrophoresis of PCR products was conducted on 2% agarose gels.

### 2.4. Sequencing Analysis

PCR products obtained in genotyping analysis were gel-purified using the QIAquick Gel extraction kit (QIAGEN, Hilden, Germany) according to the standard protocol. The purified PCR products were sequenced with an oligonucleotide primer (5′-TTCTAGTAGATAGTCCTG GTACCCAAC-3′) and Big Dye Terminator v3.1 Cycle Sequencing Kit on an ABI PRIsm 3130 Genetic Analyzer (Applied Biosystem, Thermo Fisher Scientific). *MGST2* sequencing was analyzed according to the NCBI Reference Sequence (NM_174995.3).

### 2.5. Establishment of KO Homozygous MGST2^−/−^ Mouse Lines for the MGST2 Gene

Heterozygous F1 mice were generated by mating the heterozygous G0 mouse lines (#5, #11) and wild-type C57BL/6J. KO heterozygous *MGST2*^+/−^ mouse lines were maintained by outcrossing heterozygous male mice with WT females for 3 generations. The KO homozygous *MGST2*^−/−^ mouse lines were maintained in subsequent generations by crossing heterozygous siblings.

### 2.6. Magnetic Resonance Imaging Analysis

In the pilot study, the axis angle and eye volume of ocular morphology were determined in WT and heterozygous *MGST2*^+/−^ mice. We conducted measurements on the left eye (*n* = 4, each), right eye (*n* = 4, each), and both eyes (*n* = 8) of the following pairs: F1 (one pair, 8-month-old, males), F1 (one pair, 7-month-old, males), F2 (one pair, 5-month-old, males), and F2 (one pair, 4-month-old, males). Detailed experimental conditions and measurements are described in our main study.

**Figure 1:**
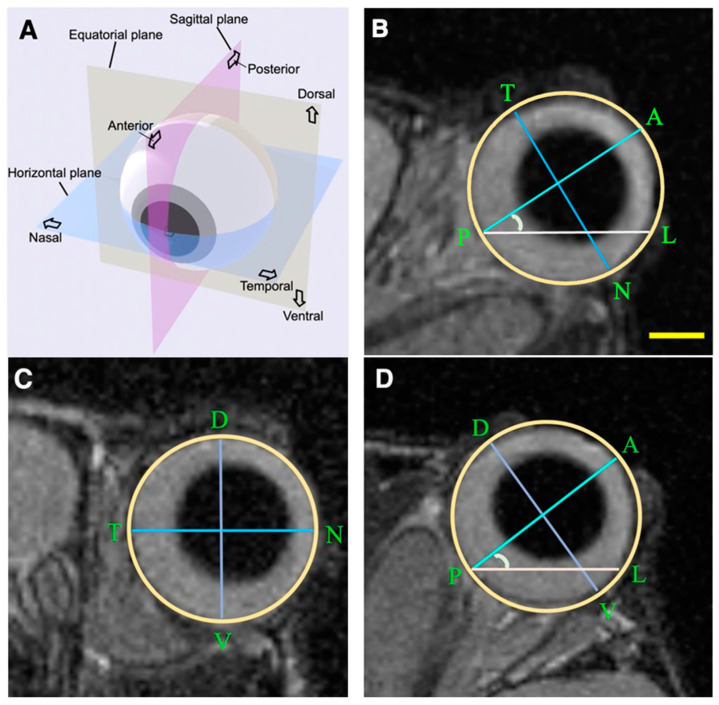
Ocular measurement via MRI. (**A**) The diagram of horizontal, equatorial, and sagittal planes of the mouse eye. (**B**) The representative image of the horizontal plane (left eye). Axis length (line AP): the distance through the center of the maximum diameter of the lens from the anterior corneal surface (point A) to the posterior pole (point P) in approximation. Eye width (line TN): the distance between the temporal (point T) and nasal (point N) of the eye, perpendicular to the axis of the eye. Axis angle (∠APL): the angle between the eye axis (line AP) and the line orthogonal (line PL) to the longitudinal fissure of the brain. The surface of the eyeball (pale yellow). (**C**) The representative image of the equatorial plane (left eye). Eye height (line DV): the distance between the dorsal (point D) and ventral (point V) of the eye. Eye width (line TN). (**D**) The representative image of the sagittal plane (left eye). Eye height (line DV). Axis length (line AP). Axis angle (∠APL). Scale bar = 1 mm.

In the main study, the ocular morphology of 5-month (20-week)-old WT mice (*n* = 5, males) and homozygous *MGST2*^−/−^ mice (*n* = 5, males) were analyzed by a high-resolution small animal MRI with a voxel size of 90 microns. The mice subjected to MRI analysis were from the F6 (one pair), F7 (two pairs), and F8 (two pairs) generations. They were acquired from heterozygous intercrosses and confirmed as WT and homozygous *MGST2*^−/−^ mice, separately. Comparisons were made between the WT mice (*n* = 5) and homozygous *MGST2*^−/−^ mice (*n* = 5). Each mouse was positioned in a prone posture on an MRI scaffold after anesthesia using a 4% concentration of isoflurane [21,22] mixed with air. The vital signs of the mice, including respiratory rate and body temperature, were carefully monitored throughout the MRI procedure to ensure that they were well-being and stable throughout the imaging session. We conducted MRI data acquisition using a 4.7 T small animal MRI system (BioSpec, Bruker BioSpin, Ettlingen, Germany) equipped with a 1H transmit–receive volume coil with an inner diameter of 23 mm. The imaging sequence (horizontally acquired) was T2-weighted 3D-Turbo rapid acquisition with relaxation enhancement (TurboRARE). To obtain a complete view of the eyeball, the following imaging parameters were established: a repetition time of 1800 ms; an echo time of 60 ms; an echo train length of 13; a slice thickness of 0.09 mm, comprising 91 slices; a field of view of 20 × 20 mm; and a matrix size of 222 × 278. The multiplanar reconstruction tool (3D MAP of the Horos software (version 3.0, The Horos Project)) was used to generate horizontal, equatorial, and sagittal planes. To execute the reconstruction process, the horizontal plane was utilized as a reference plane. In the initial step, we selected an image that depicted the eye axis, spanning from the anterior corneal surface through the center of the maximum diameter of the lens approximately to the posterior pole. Subsequently, a sagittal plane was defined perpendicular to the horizontal plane that passed through the eye axis in thorough visualization analysis. The plane perpendicular to both the horizontal and sagittal planes was designated as the equatorial plane (Figure 1A). The ocular dimensions were measured on the horizontal, equatorial, and sagittal images with ImageJ software (version 1.53K, NIH, Bethesda, MD, USA). 

To perform morphometric analyses of the eyeball, measurement parameters were defined as follows. The axis length and axis angle were measured from the image through the horizontal plane and sagittal plane of the eye (Figure 1B,D). The length of eye axis (line AP) was recorded as the distance spanning from the anterior corneal surface (point A) through the center of the maximum diameter of the lens to the posterior pole (point P) in approximation. Axis angle (∠APL) was measured as the angle between the eye axis (line AP) and the line orthogonal to the longitudinal fissure of the brain (line PL). Eye width (line TN) was measured as the distance between the temporal (point T) and nasal (point N) of the eye, perpendicular to the axis of the eye on horizontal plane and equatorial plane (Figure 1B,C). The volume was measured from regions of interest (ROIs) set on slices in the horizontal plane. Specifically, it was calculated by converting pixel counts from the ROIs of the entire eye, based on the image resolution. Eye height (line DV) was defined as the distance between the dorsal (point D) and ventral (point V) of the eye on the sagittal plane, which was perpendicular to the eye axis, and equatorial plane (Figure 1C,D). The cross-sectional area of the eyeball was measured in the horizontal plane, equatorial plane, and sagittal plane, correspondingly (Figure 1B–D). All measurement parameters were separately assessed on ImageJ for the left eye (*n* = 5, each), right eye (*n* = 5, each), and both eyes (*n* = 10). 

### 2.7. Statistical Analysis

By considering the contribution of both eyes in binocular vision and also the possibility of magnetic field inhomogeneity between both eyes in MRI (see Section 4), we decided to combine the data from both eyes to eliminate possible variations between the left and right eyes, as well as to ensure the consistency and reliability of the acquired data. Statistical analysis for measurements of the relevant parameters was conducted by SPSS for Mac (version 28.0). In Student’s *t*-test, *p* < 0.05 was considered significant, and all data were presented as mean ± standard deviation (SD).

## 3. Results

### 3.1. Generation of an MGST2 KO Mouse

**Figure 2:**
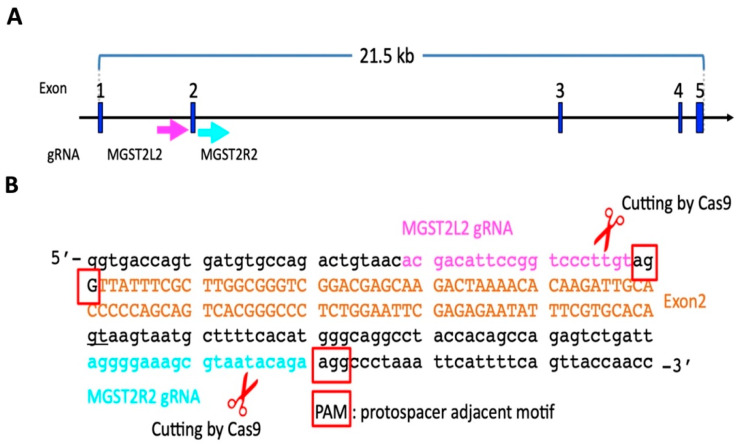
CRISPR/Cas9-mediated generation of a *MGST2* KO mouse model. (**A**) Schematic diagram of gRNA targeting sites at the *MGST2* gene in mice. Exons are represented by blue boxes. The gRNA MGST2L2 is marked in pink, and the gRNA MGST2R2 is marked in fluorescent blue. (**B**) Genomic sequence around *MGST2* exon 2 with cut by Cas9/gRNA complex. The protospacer adjacent motif (PAM) sequence is labeled in red frames. Exon 2 sequences are represented in brown.

**Figure 3:**
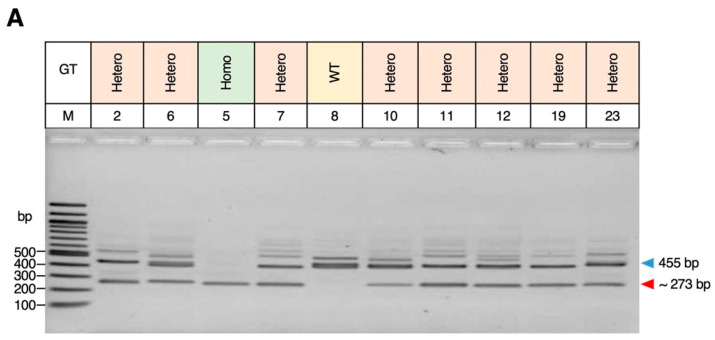

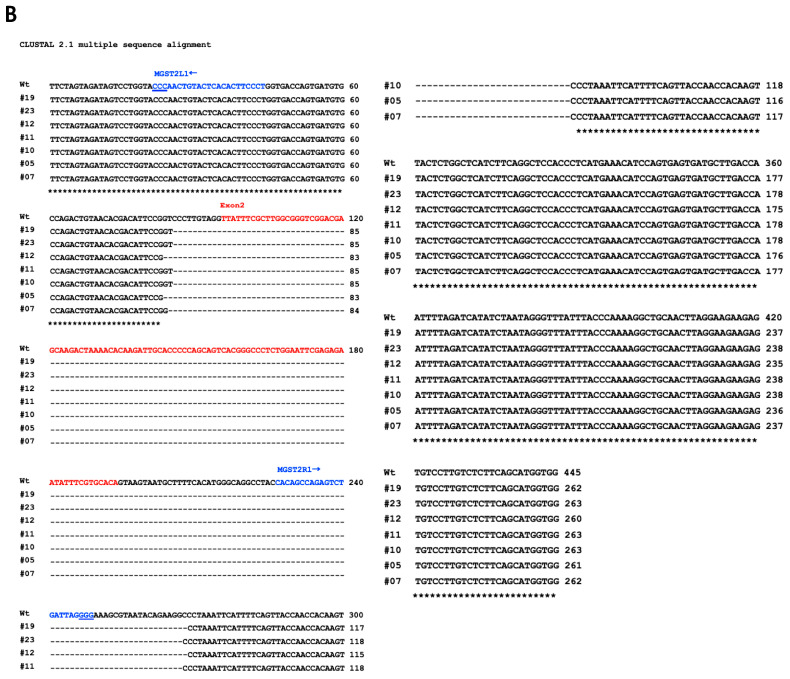
The genotypes of the G0 mice in CRISPR-Cas9 targeting the *MGST2* gene. A total of 10 mice from the total 23 mice (survival) in the G0 generation: #2, #6, #5, #7, #8, #10, #11, #12, #19, and #23 for genotyping. (**A**) Electrophoresis of *MGST2* gene PCR products in G0 generation. Amplicons of 455 bp (WT) and ≈273 bp (mutant) were obtained. M: marker; bp: base-pair; GT: genotyping; Hetero: heterozygous; Homo: homozygous. (**B**) The PCR products of the above genotypes were pick up for DNA sequencing analysis. #19 (♀) mouse shows deletion with 183 bp, #23 (♀) deletion with 182 bp, #12 (♂) deletion with 185 bp, #11 (♂) deletion with 182 bp, #10 (♂) deletion with 182 bp, #5 (♂) deletion with 184 bp, #7 (♂) deletion with 183 bp; exon 2 sequence is labeled in red; gRNA (MGST2L2, MGST2R2) is represented by blue. Asterisks indicate positions where nucleotides are identical in all sequences.

In order to determine what role the *MGST2* gene would play in the development of eye morphology, the *MGST2* KO mice were generated using the CRISPR/Cas9 system. In the sequential electroporation with gRNAs (Mgst2L2 and Mgst2R2) to target DNA recognition sites (Figure 2A,B), the yield of mice (born/treated zygotes) was 15% (23/149), and the KO rate was 39% (9/23). Herein, 10 mice were selected from the total of 23 mice in the G0 generation (#2, #5, #6, #7, #8, #10, #11, #12, #19, and #23 mouse) for DNA extraction, PCR amplification, and agarose gel electrophoresis. The gel electrophoresis demonstrated that only mouse #8 had the WT (455 bp) genotype, while the remaining mice displayed the KO (≈273 bp) genotype, leading to preliminary confirmation of *MGST2* gene mutations (Figure 3A). DNA sequencing was performed on PCR products obtained from mice #5 (♂), #7 (♂), #10 (♂), #11 (♂), #12 (♂), #19 (♀), and #23 (♀) of the above samples. The sequencing results confirmed that these samples were positive for KO in G0 generation mice with *MGST2* exon 2 deleted and thus proved that the *MGST2* gene had been knocked out (Figure 3B).

### 3.2. Magnetic Resonance Imaging of the Mice Eyes

In the pilot study, the ocular morphology was measured in WT mice and heterozygous *MGST2*^+/−^ mice (Table 1). The axis angle and eye volume showed no significant difference between the heterozygous *MGST2*^+/−^ mice and the WT mice. Thus, we designed the main study to compare the homozygous *MGST2*^−/−^ mice and the WT mice as follows.

**Figure 4:**
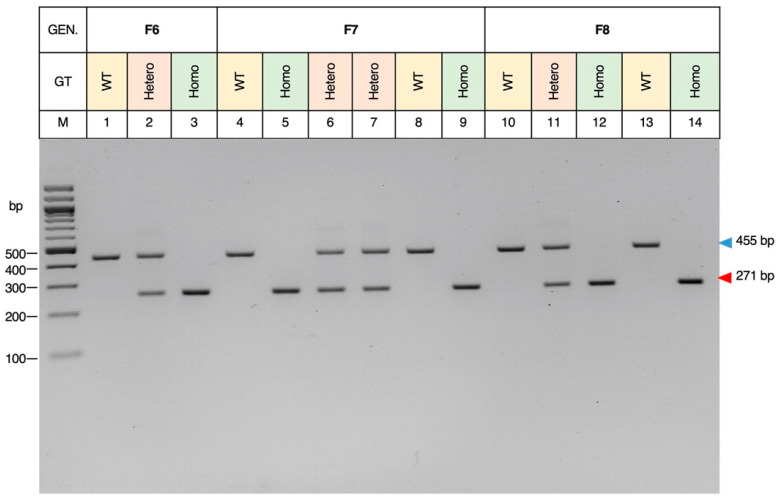
The genotypes in the F6, F7, and F8 generation mice of the KO mouse line #5. The WT and homozygous genotype were selected as samples for the MRI experiment. One pair of F6 generation, two pairs of F7 generation, and two pairs of F8 generation in the total of 10 mice were used for MRI analysis and comparison. M: marker; bp: base-pair; WT: 455 bp, KO: 271 bp; GT: genotype; GEN.: generation; Hetero: heterozygous; Homo: homozygous.

**Figure 5:**
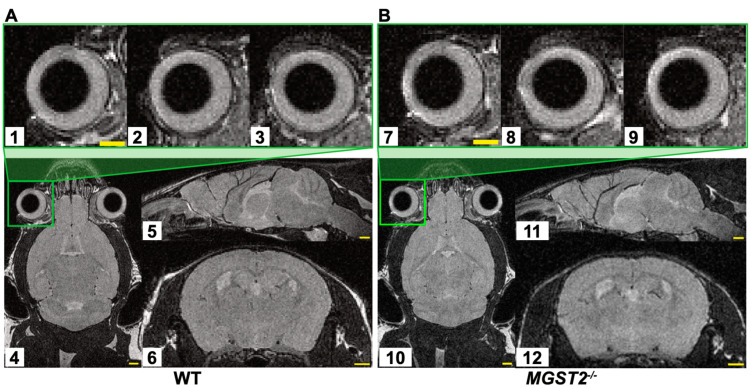
Representative MRI images of WT (**A**) and homozygous *MGST2*^−/−^ (**B**) mice. No remarkable alterations in the anatomical overview of the eye and brain. The upper panels show close-up views of the right eye for WT (1, 2, 3) and homozygous *MGST2*^−/−^ (7, 8, 9) mice in the horizontal plane, coronal plane, and sagittal plane. The lower panels show the brain for WT and homozygous *MGST2*^−/−^ mice in horizontal (4, 10), sagittal (5, 11), and coronal planes (6, 12) constructed by multi-planer reconstruction. Scale bar = 1 mm.

Figure 4 shows representative genotypes of F6, F7, and F8 mice (WTs and homozygotes), which were the offspring of a mating between heterozygotes of the KO mouse line #5 and used for the MRI experiment. In the anatomical overview, MRI images of the eyes and brains of homozygous *MGST2*^−/−^ mice and heterozygous *MGST2*^+/−^ mice were not substantially altered morphologically compared to WT mice, either in the horizontal, coronal, or sagittal planes in Horos software (Figure 5).

**Figure 6:**
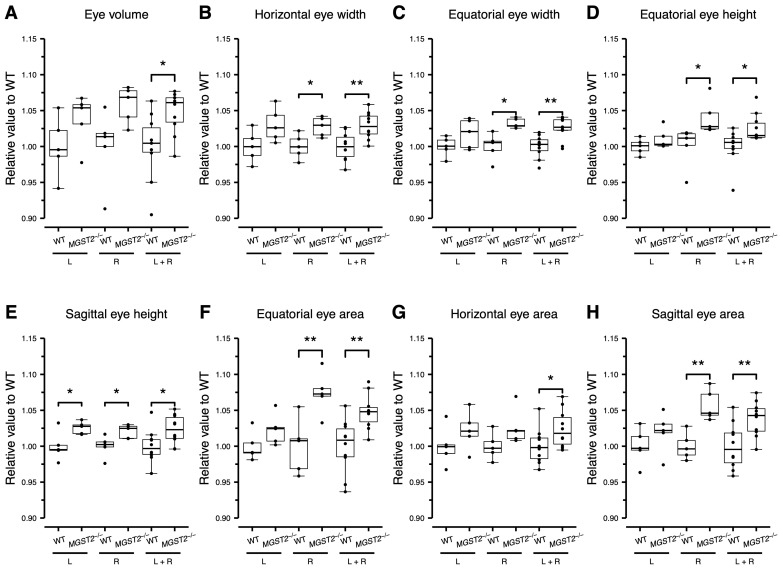
Eye morphometric analysis. Box-and-whisker and dot plots of relative values of homozygous *MGST2*^−/−^ to WT for each measured parameter. The standardization of each panel was calculated by the WT average. The vertical axis represents relative values, and the horizontal axis represents the left eye (L, *n* = 5, each), right eye (R, *n* = 5, each), and combined left and right eye (L + R, *n* = 10) of WT and homozygous *MGST2*^−/−^ samples, correspondingly. The lower hinge, the central bold line, and the upper hinge represent the first quartile, the median, and the third quartile, respectively. Whiskers extend to the most extreme data points within 1.5 times the interquartile range. Data points outside the range of the whiskers are denoted as outliers. *: *p* < 0.05, **: *p* < 0.01.

To examine whether the KO of the *MGST2* gene would cause eye misalignment, the axis angle was measured in horizontal and sagittal planes of WT and homozygous *MGST2*^−/−^ mice, as shown in Figure 1B,D. There were no significant differences in the axis angles of left, right, or both eyes (Table 2 A). Next, the eye volumes of mice were compared to determine whether the *MGST2* gene could affect eye morphology. The analytic calculations showed that the eye volumes of homozygous *MGST2*^−/−^ mice were increased by 5.6%, compared with those of WT mice (Table 2 A, Figure 6A). 

To understand the cause of the eye volume increase, the eye axis length, eye height, and eye width were measured. As the result of measurements, homozygous *MGST2*^−/−^ mice showed their increased eye width in both the horizontal and equatorial planes (Figure 1B,C) by 3% (Table 2 A, Figure 6B,C) and their increased eye height in equatorial and sagittal planes (Figure 1C,D) by 2.4% (Table 2 B, Figure 6D,E), compared with WT mice. In contrast, the length of eye axis was not different between homozygous *MGST2*^−/−^ mice and WT mice (Table 2 A). As the width and height of the eyes increased, we expected that the area on the equatorial plane would change as well. The subsequent analysis showed that the area on the equatorial plane of homozygous *MGST2*^−/−^ mice was 4.6% larger than that of WT mice (Table 2 B, Figure 6F). To ensure the reliability of the results, the area on the horizontal and sagittal planes was measured. The results showed that the area on the horizontal plane was greater than 2.4% and that the area on the sagittal plane was greater than 3.6% in the homozygous *MGST2*^−/−^ mice, compared with the WT mice (Table 2 A, Figure 6G,H).

Overall, there were no significant variations in axis length and axis angle between homozygous *MGST2*^−/−^ mice and WT mice. However, the morphometric analyses showed that the eye height, width, and volume in homozygous *MGST2*^−/−^ mice were significantly greater than those of WT mice, indicating that the eyes of *MGST2*^−/−^ mice were enlarged.

## 4. Discussion

The aim of this study was to look for the role of the *MGST2* gene as a candidate strabismus-susceptibility gene in eye development, eye alignment, and overall eye morphology as possible background for the development of comitant strabismus. We measured the axis length, axis angle, width, height, area, and volume of the eyes by high-resolution small animal MRI on the *MGST2* gene knockout model. The results showed that the eyes of the homozygous *MGST2*^−/−^ mice were larger than those of the WT mice, although there was no change in axis length and axis angle.

The no significant differences in axis angle and axis length measurements between WT and homozygous *MGST2*^−/−^ mice suggest that *MGST2* might not play a substantial role in determining eye alignment. The significant increase in eye volume in homozygous *MGST2*^−/−^ mice compared with WT mice indicates that the *MGST2* gene may influence overall eye size. The observed changes in eye height and width contributed to this increase in eye volume. The eye area had differences in all three planes but was largest in the equatorial plane, followed by the sagittal and horizontal planes, highlighting size differences in the spatial dimensions of the eye. 

*MGST2* plays a key role in the synthesis of leukotriene C4 (LTC4), an important lipid mediator, which binds to its internalized receptor and triggers the translocation of NADPH oxidase 4 (NOX4) to the nucleus and perinuclear region of the cell, resulting in the generation of reactive oxygen species (ROS), oxidative damage to nuclear DNA, and induction of apoptosis and necrosis. In contrast, ER-stress-induced cell death was shown to be reduced in the kidney of *MGST2* KO homozygous *MGST2*^−/−^ mice [18,23]. Although ER-stress-induced apoptosis mediated by *MGST2* has not been reported in eyes, the absence of *MGST2* may prevent cell death that should occur during eye development and differentiation, resulting in increased eye volume. Under the circumstances, apoptosis mechanism in *MGST2* KO heterozygous *MGST2*^+/−^ mice remains unknown. The reason why the heterozygous *MGST2*^+/−^ mice in this study did not show significant changes in the axis angle and eye volume would be possibly because they possess at least one normal copy of the *MGST2* gene, which is sufficient to maintain normal development and differentiation of the eye structure.

Extrapolating these findings to the broader context of ocular morphology, it is important to note that various factors can contribute to the shape of the eye, which would be influenced by alterations in the anatomical structures of the eye, including the cornea, lens, retina, and sclera [24,25]. In this study, the result of eyeball enlargement was obtained by measuring the volume of the entire eyeball surface. There may be abnormalities in the attachment of the extraocular muscle to the sclera on the changed dimension of the eyeball surface. In that case, eye movement would be changed to some extent in the direction of action of the relevant muscles, which might lead to the development of strabismus [26]. As a methodological limitation in this study, the imaging of certain structures and tissues of the eye can be indeed dependent on the MRI conditions. Small changes in these structures may not be observable, even at higher resolution. The use of MRI contrast agents may help overcome this limitation in our future observations. MRI contrast agents are substances that can enhance the contrast and clarity of images and provide greater detail of tissue structure [27]. 

In cases such as myopia and hyperopia, the impact on the axis length of the eye potentially results in changes in its overall shape [28]. Indeed, it was found that most (95.7%) myopic eyes showed oblate or elongated eyeball shapes, that hyperopic eyes were predominantly oblate or broadly shaped, and that astigmatic eyes were mostly oblate or spherical [29]. The other example of a disease that contributes to such morphological changes of the eye would be glaucoma, which can cause internal expansion of the eyeball’s structure and result in an increased eyeball volume in a group of patients by elevated intraocular pressure [30]. Additionally, proptosis, or exophthalmos, is another example where changes in eye appearance, including eye enlargement, are observed without necessarily affecting the axis length or axis angle of eye [31]. Certain congenital or acquired eye disorders as well as some neurologic disorders such as elevated intracranial pressure can also lead to changes in the morphology of the eye [32,33,34,35]. These findings highlight the complexity and multifaceted nature of the factors that influence eye morphology. Although the current results in our animal model may have limitations to be directly applied to comitant strabismus, a broader understanding of the potential causes of changes in eye morphology or eye development would give a hint for the development of strabismus.

As a methodological limitation, there may be image distortion in this experiment. Because the uniformity of the main magnetic field degrades with distance from the isocenter, image distortion appears at the edges of the imageable area [36,37]. The image distortion may have caused each of the left and right eyes to be stretched or contracted on the image. Since the mice in the two groups for comparison were almost at the same age and were aligned as much as possible in terms of MRI positioning, the effect of the image distortion would be approximately equivalent among the mice. Additionally, to avoid any image distortion that may occur in the analysis, only the same eye (either the right eye or left eye) was used for comparison between the data of WT mice and *MGST2* KO homozygous *MGST2*^−/−^ mice. Furthermore, the TurboRARE sequence employed in this study is most insensitive to inhomogeneity of main magnetic fields among MRI pulse sequences, and thus the image distortion is minimal, and bias is limited. We also considered the participation of both eyes in binocular visual integration as well as the symmetry of the anatomical structures, and thus we decided to combine the data from the left and right eyes for analysis to reduce individual differences and to improve the reliability of the data. In this context, any existing bias would minimally impact the interpretation of morphological differences between WT and *MGST2* KO homozygous *MGST2*^−/−^ mice. At present, it remains to be still challenging to achieve complete uniformity in the main magnetic field of actual MRI equipment, and the image distortion cannot be avoided completely. Future studies could enhance the measurement accuracy by acquiring a main magnetic field map and by correcting image distortions in post-processing [38].

A major limitation of this study is the question of whether a mouse model would be suitable for the analysis of a clinical disease such as comitant strabismus in humans. Humans have a macular structure of the retina with a high resolution of vision. The eyes in humans are located at the front to have a wide range of the visual field, overlapping in both eyes to have binocularity. The human eyes also have a specific feature to show the cornea in the visible background of the conjunctiva to make apparent the eye position and hence the eye alignment. In contrast, mice have the eyes on each side of the head, and only their cornea, but not their conjunctiva, is visible between the eyelids, leading to difficulty in identifying the eye position and alignment. Even with these differences between the humans and mice, the mice do have binocularity with a smaller range of overlapping visual fields of both eyes. Under the circumstances, the mouse model would provide a meaningful hit or key for understanding the mechanism of comitant strabismus in humans. The extraocular muscles that attach to the eyeball would be a candidate for morphological changes in comitant strabismus [39,40]. In the case that the eyeball has a different shape, the torque exerted by the extraocular muscles would be changed, and thus the eye position would be shifted to develop comitant strabismus.

## 5. Conclusions

*MGST2* KO homozygous *MGST2*^−/−^ mice showed significantly larger height, width, and volume of the eyeballs than those of WT mice, indicating that the eyes of *MGST2* KO homozygous *MGST2*^−/−^ mice were significantly enlarged. These morphological changes may be an underlying factor in the subgroup of strabismus and would provide new insights into the pathogenesis of eye abnormalities. Further research, such as molecular pathways and cell signaling, is needed to understand the specific mechanisms. A better understanding of the *MGST2* gene’s association with eye abnormalities may lead to improved treatments and diagnostic tools and thus to the advance in the field of visual health.

## Figures and Tables

**Table 1:** The quantitative analysis data of eye morphology in WT and heterozygous *MGST2*^+/−^ mice across different-age pairs.

	Horizontal Plane						Sagittal Plane		
Genotype	WT	*MGST2^+/^* ^−^	*p*	WT	*MGST2^+/^* ^−^	*p*	WT	*MGST2^+/^* ^−^	*p*
	Volume (mm^3^)			Axis Angle (°)			Axis Angle (°)		
L	21.88 ± 1.99	22.2 ± 0.97	0.63	28.42 ± 1.9	31.75 ± 4.5	0.24	33.12 ± 3.3	32.16 ± 2.3	0.46
R	22.25 ± 1.81	22.53 ± 1.6	0.73	28.87 ± 4.1	28.73 ± 4.9	0.95	33.76 ± 2.9	32.75 ± 3.3	0.65
L + R	22.13 ± 1.9	22.2 ± 1.3	0.51	28.64 ± 3.4	27.68 ± 5.3	0.33	33.44 ± 2.9	32.46 ± 2.7	0.52

We conducted measurements on the following pairs: F1 (one pair, 8-month-old, males), F1 (one pair, 7-month-old, males), F2 (one pair, 5-month-old, males), and F2 (one pair, 4-month-old, males). The data for the left eye (L, *n* = 4, each), right eye (R, *n* = 4, each), and both eyes (L + R, *n* = 8) are presented as mean ± SD. The measurements were taken on two different planes: the horizontal plane and the sagittal plane. The following parameters were measured: volume and axis angle on the horizontal plane, as well as the axis angle on the sagittal plane. Statistical tests were performed on L, R, and L + R. *p-*values were calculated using the paired-sample *t*-test.

**Table 2:** The quantitative analysis data of eye morphology in WT and homozygous *MGST2*^−/−^ mice.

	**Horizontal Plane (A)**								
**Genotype**	**WT**	***MGST2* ^−/−^**	***p***	**WT**	***MGST2* ^−/−^**	***p***	**WT**	***MGST2* ^−/−^**	***p***
	**Axis Angle (°)**			**Volume (mm^3^)**			**Width (mm)**		
L	23.06 ± 1.54	23.19 ± 1.52	0.97	22.3 ± 0.68	22.3 ± 0.68	0.17	3.29 ± 0.07	3.39 ± 0.07	0.07
R	23.24 ± 3.49	23.5 ± 0.83	0.88	21.9 ± 1.01	23.8 ± 0.24	0.07	3.31 ± 0.05	3.41 ± 0.04	0.02
L + R	23.16 ± 2.57	24.16 ± 1.53	0.92	22.1 ± 0.97	23.4 ± 1.33	0.01	3.3 ± 0.05	3.40 ± 0.06	0.005
**Genotype**	**WT**	***MGST2* ^−/−^**	***p***	**WT**	***MGST2* ^−/−^**	***p***			
	**Axis Length (mm)**			**Area (mm^2^)**					
L	3.34 ± 0.03	3.33 ± 0.05	0.86	9.32 ± 0.25	9.53 ± 0.25	0.23			
R	3.34 ± 0.04	3.32 ± 0.04	0.61	9.14 ± 0.17	9.38 ± 0.23	0.1			
L + R	3.33 ± 0.03	3.31 ± 0.03	0.64	9.23 ± 0.23	9.45 ± 0.24	0.046			
	**Equatorial Plane (B)**								
**Genotype**	**WT**	***MGST2* ^−/−^**	***p***	**WT**	***MGST2* ^−/−^**	***p***	**WT**	***MGST2* ^−/−^**	***p***
	**Width (mm)**			**Height (mm)**			**Area (mm^2^)**		
L	3.23 ± 0.04	3.29 ± 0.06	0.15	3.18 ± 0.03	3.22 ± 0.04	0.22	8.96 ± 0.18	9.17 ± 0.19	0.12
R	3.22 ± 0.06	3.32 ± 0.02	0.01	3.11 ± 0.08	3.24 ± 0.07	0.04	8.56 ± 0.33	9.19 ± 0.25	0.001
L + R	3.22 ± 0.05	3.10 ± 0.05	0.002	3.15 ± 0.08	3.23 ± 0.06	0.02	8.76 ± 0.33	9.18 ± 0.21	0.003
	**Sagittal Plane (C)**								
**Genotype**	**WT**	***MGST2* ^−/−^**	***p***	**WT**	***MGST2* ^−/−^**	***p***	**WT**	***MGST2* ^−/−^**	***p***
	**Axis Angle (°)**			**Axis Length (mm)**			**Height (mm)**		
L	22.8 ± 3.72	26.4 ± 3.99	0.17	3.39 ± 0.03	3.39 ± 0.06	0.95	3.29 ± 0.06	3.38 ± 0.03	0.045
R	23.4 ± 4.26	21.8 ± 3.77	0.53	3.37 ± 0.04	3.34 ± 0.03	0.17	3.00 ± 0.2	3.27 ± 0.25	0.034
L + R	23.1 ± 3.78	24.1 ± 4.39	0.59	3.38 ± 0.04	3.36 ± 0.05	0.46	3.26 ± 0.07	3.34 ± 0.06	0.025
	**Area (mm^2^)**								
L	8.83 ± 0.22	9.00 ± 0.25	0.29						
R	8.45 ± 0.16	8.93 ± 0.18	0.002						
L + R	8.64 ± 0.27	8.97 ± 0.21	0.008						

We conducted measurements on the following pairs: F6 (one pair, 5-month-old, males), F7 (two pairs, 5-month-old, males), and F8 (two pairs, 5-month-old, males). The data for the left eye (L, *n* = 5, each), right eye (R, *n* = 5, each), and both eyes (L + R, *n* = 10) are presented as mean ± SD. The measurements were taken on three different planes: the horizontal plane, the equatorial plane, and the sagittal plane. The following parameters were measured: axis angle, volume, width, axis length, and area on the horizontal plane (A); width, height, and area on the equatorial plane (B); and axis angle, axis length, height, and area on the sagittal plane (C). The sample size for each measurement was 5 for each eye and 10 for both eyes. Statistical tests were performed on L, R, and L + R. *p*-values were calculated using Student’s *t*-tests.

## Data Availability

The original datasheet, created and analyzed in the current study, is available from the corresponding author upon reasonable request.
